# Thermal conductivity of an ultracold Fermi gas in the BCS-BEC crossover

**DOI:** 10.1038/s41598-020-79010-w

**Published:** 2021-01-13

**Authors:** Hang Zhou, Yongli Ma

**Affiliations:** State Key Laboratory of Surface Physics and Department of Physics, Fudan University, Shanghai, 200433 China

**Keywords:** Ultracold gases, Thermodynamics

## Abstract

Recent experiments on sound waves in a unitary Fermi gas reveal many transport properties about strongly interacting fermions. Sound propagates through the coupling of momentum and heat transport, and attenuates strongly with the presence of a phase transition. In this work, focusing on the temperature regimes near and below the superfluid critical temperature $T_c$ in the BCS-BEC crossover, we present a Kubo-based microscopic calculation of thermal conductivity $\kappa$, which has not attracted much attention compared to the shear viscosity. Our approach primarily addresses the contributions of the fermionic quasiparticles to thermal transport and our results return to the kinetic descriptions at high temperatures. $\kappa$ drops upon crossing the pseudogap temperature $T^*$, and its temperature dependence changes below $T_c$. The drops become more pronounced on the weakly coupled BCS side, where the Pauli blocking causes the upturn of $\kappa$ above $T^*$. Our calculations fit well with the sound measurement on the damping rate.

## Introduction

Ultracold Fermi gases via Feshbach resonance could undergo a smooth crossover from the Bardeen-Cooper-Schrieffer (BCS) state of weakly-correlated pairs of fermions to the Bose-Einstein condensation (BEC) of diatomic molecules. In the unitary limit, the *s*-wave scattering length $a_f$ diverges. The tunable attractions arouse preformed pairs in the normal phase roughly at temperature $T^*$, known as the ‘pseudogap state’. The condensation of pairs occurs below $T_c$ in the ordered phase. Fermi gas with this highly controllable advantage and clean environment provides a model system to study physical properties in condensed matter and nuclear physics^1–3^.

In the last decade, the viscous transport of unitary Fermi gases has drawn great attention, as it is a nearly perfect fluid similar to the quark-gluon plasma^4^. Measurements and theories have found anomalously small ratios of shear viscosity $\eta$ to entropy density *s*^5–11^, which are remarkably close to the conjectured universal lower^12^ and upper bounds^13^, $1/(4\pi )\leqslant \eta /s\lesssim v^2T\tau _\eta$, with $\hbar =k_B=1$ throughout the paper, the temperature *T*, the typical velocity scale *v* and viscous thermalization time $\tau _\eta$. These high precision determinations of $\eta$ could be served as key inputs to further transport investigations, such as to deduce the thermal conductivity $\kappa$ from sound waves^14^. Recent experiments on ultracold Fermi gases have allowed local transport measurements in nearly uniform densities^15^, which enable direct comparison between theory and experiment. Several experiments have explored the damping rate of sound waves from the density response function^14,16,17^, which contains both the contributions of momentum and temperature gradients related to the shear and bulk viscosities and thermal conductivity, respectively. These experiments show that the unitary Fermi gas shares some universal but not exactly similar characteristics with liquid $^4\mathrm{He}$ and $^3\mathrm{He}$, by respectively probing the collisonless and hydrodynamic regimes^14,16^. Meanwhile, theoretical works have accessed into the lower temperature regions. The damping rates of the collective excitations near zero temperatures have been studied by considering various damping mechanisms of phonons with different curvature of dispersion relation, such as the four-phonons Landau-Khalatnikov process, three-phonons Beliaev process and the inelastic process between phonons and fermions^18–20^. Based on the two-fluid hydrodynamics, two-sound waves descriptions related to the general transport coefficients have also been developed^21,22^. However, due to the lack of investigations on $\kappa$, these predictions in superfluid have to be made using the values of $\kappa$ either from the high-*T* extrapolation of Boltzmann results^22,23^ or from the low-*T* calculations by phonons that are also at the kinetic level^21,23^. A kinetic description of transport coefficients works well with long-lived quasiparticle excitations, which in a strongly correlated Fermi gas fails below $T^*$ with the appearance of additional bosonic degrees of freedom that are responsible for the gap structures of excitation spectra^2,3^. It is an urgent task to establish a microscopic calculation of $\kappa$ in the superfluid and pseudogap states.

Since the condensed pairs do not carry entropy and therefore do not contribute to the thermal transport, $\kappa$ is a well-tailored probe of the low-energy excitation properties in many important superconducting materials. There are various effects in a material that may be sensitive to heat transport, such as the elastic or inelastic scatterings among electrons, phonons, impurities or other exotic degrees of freedom. Circumstances are much simpler in a clean BCS-BEC crossover scenario, especially in the superfluid phase: the non-condensed Fermi pairs together with the unpaired fermions form the thermally excited quasiparticles of the system. Analysis of the lifetimes of the two quasiparticles shows that the scattering between fermions is the dominant relaxation mechanism of the system^11^.

In this paper, mainly focusing on the temperatures near and below $T_c$, we investigate $\kappa$ from the Kubo formulas on current-current correlation functions. Although various versions of *t*-matrix theories have been considered at the normal states^24^, it is still difficult to calculate the transport coefficients microscopically in the superfluid phase. We are based on the microscopic pseudogap theory^3^, which involves some simplifications by decomposing the self energy into the pseudogap contribution associated with small-momentum pairs and superfluid contribution with condensed pairs. This theory has the same asymmetric form of *t*-matrix approach as the mean-field theory, that ensures the agreement of our calculations of $\kappa$ with the results by Kadanoff and Martin^25^ at BCS limit. Meanwhile, the kinetic results^26^ can be directly derived from our Kubo-based calculations at high temperatures. We show drops of $\kappa$ upon crossing below $T^*$, where preformed pairs bring the loss of thermal carriers. The fluctuations of these pairs lead to different temperature dependencies around $T_c$, which become non-monotonic near the unitary limit. Below $T_c$, the estimate of the thermal relaxation rate $\tau ^{-1}$ is smaller than the characteristic energy scales of the system, which implies that the system is in a weak dissipation region. So we combine our results of $\kappa$ with our previous work on $\eta$ to obtain the damping rate $\Gamma$ of sound, and the results fit well with experiments in collisionless regime.

## Results

### Kubo formula for the thermal conductivity

In the BCS-BEC crossover scenario, the two-component Fermi gas is described by the Hamiltonian with zero-range interactions

1 $$\begin{aligned} \hat{H}=\sum _{{\mathbf{k}}\sigma }\xi _{\mathbf{k}} \hat{c}_{{\mathbf{k}}\sigma }^\dag \hat{c}_{{\mathbf{k}}\sigma } +\frac{g}{2}\sum _{{\mathbf{k}}{\mathbf{k}}'{\mathbf{q}}\sigma \sigma '} \hat{c}_{{\mathbf{k}}+{\mathbf{q}}\sigma }^\dag \hat{c}_{{\mathbf{k}}'-{\mathbf{q}} \sigma '}^\dag \hat{c}_{{\mathbf{k}}'\sigma '}\hat{c}_{{\mathbf{k}}\sigma }. \end{aligned}$$

Here $\xi _{\mathbf{k}}={\mathbf{k}}^2/2m-\mu$ is the dispersion measured from the chemical potential $\mu$, *m* is the atomic mass and $\hat{c}_{{\mathbf{k}}\sigma }^\dag (\hat{c}_{{\mathbf{k}}\sigma })$ is the fermion creation(annihilation) operator with the pseudospin $\sigma =\uparrow ,\downarrow$. *g* is the bare *s*-wave interaction strength associated with the tunable scattering length $a_f$, given by $\frac{1}{g}=\frac{m}{4\pi a_f}-\sum _{{\mathbf{k}}}\frac{m}{{\mathbf{k}}^2}$.

A neutral Fermi atomic system contains particle (${\mathbf{J}}_1$) and heat (${\mathbf{J}}_2$) currents, their operators can be expressed in terms of the above Hamiltonian as^27,28^

2 $$\begin{aligned} \begin{aligned} {{\mathbf{j}}}_1({\mathbf{q}},t)=&\frac{1}{2m}\sum _{{\mathbf{k}}\sigma }(2{\mathbf{k}}+{\mathbf{q}}) \hat{c}_{{\mathbf{k}}\sigma }^\dag (t)\hat{c}_{{\mathbf{k}}+{\mathbf{q}}\sigma }(t),\\ {\mathbf{j}}_2({\mathbf{q}},t)=&\frac{1}{2m}\sum _{{\mathbf{k}}\sigma } [{\mathbf{k}}\xi _{\mathbf{k}}+{\mathbf{q}}+({\mathbf{k}}+{\mathbf{q}})\xi _{\mathbf{k}}] \hat{c}_{{\mathbf{k}}\sigma }^\dag (t)\hat{c}_{{\mathbf{k}}+{\mathbf{q}}\sigma }(t) +g\sum _{{\mathbf{k}}{\mathbf{k}}'{\mathbf{q}}'}\frac{{\mathbf{q}}'}{m} \hat{c}_{{\mathbf{k}}+{\mathbf{q}}+{\mathbf{q}}'\uparrow }^\dag (t) \hat{c}_{{\mathbf{k}}'-{\mathbf{q}}'\downarrow }^\dag (t) \hat{c}_{{\mathbf{k}}'\downarrow }(t)\hat{c}_{{\mathbf{k}}\uparrow }(t). \end{aligned} \end{aligned}$$

Under linear response assumption, the currents flow directly proportional to the external forces ${\mathbf{X}}_j$: ${\mathbf{J}}_i=\sum _{j=1}^2L_{ij}{\mathbf{X}}_j$. We take a specific form of concentration gradient ${\mathbf{X}}_1=-\nabla (\frac{\mu }{T})$ and temperature gradient ${\mathbf{X}}_2=\nabla (\frac{1}{T})$, so that the Onsager relation holds as $L_{12}=L_{21}$. The four coefficients $L_{ij}$ ($i,j=1,2$) are related to the general transport coefficients, such as the particle conductivity $\sigma _N=L_{11}/T$ and thermopower $\varsigma =L_{12}/(TL_{11})$. Thermal conductivity $\kappa$ is usually defined as ${\mathbf{J}}_2=-\kappa \nabla T$ and measured under the condition ${\mathbf{J}}_1=\mathbf{0}$, which immediately leads to $\kappa =\frac{1}{T^2}\left( L_{22}-\frac{L_{12}L_{21}}{L_{11}}\right)$^29^. In the Supplementary Information (SI), we give a generalized derivation of the four static coefficients $L_{ij}$ from the corresponding correlation functions within the linear response theory,

3 $$\begin{aligned} L_{ij}=-\lim _{\Omega \rightarrow 0}\frac{T}{\Omega }\lim _{{\mathbf{q}}\rightarrow 0} \mathrm{Im}\left( \frac{{\mathbf{q}}\cdot \overleftrightarrow {L}_{ij} ({\mathbf{q}},\Omega )\cdot {\mathbf{q}}}{q^2}\right) . \end{aligned}$$

Here the current-current correlation functions $\overleftrightarrow {L}_{ij}({\mathbf{q}},\Omega )$ can be obtained by the Fourier transform of the retarded correlation functions $\overleftrightarrow {L}_{ij}({\mathbf{q}},t)=\mathrm{i}\Theta (t) \langle [{\mathbf{j}}_i({\mathbf{q}},t),{\mathbf{j}}_j(-{\mathbf{q}},0)]\rangle$, where the step function $\Theta (t)$ enforces the causality and $\langle \cdots \rangle$ denotes the thermodynamic average. The current operators as well as the correlation functions can be associated with the single-particle Green’s function $G({\mathbf{k}},t)=-{\mathrm{i}}\Theta (t)\langle [{\hat{c}}_{{\textbf{k}} \sigma }(t),{\hat{c}}_{{\textbf{k}}\sigma }^\dag (0)]\rangle$ (More details can be seen in the Supplementary Information).

In the BCS-BEC crossover, the most commonly used microscopic approaches to get the Green’s functions which incorporate pairing fluctuations are the many-body *t*-matrix theories^1^. There are at least five kinds of alternative *t*-matrix approaches that can be numerically adopted above $T_c$^24^. However, in the superfluid phase, numerical calculations remain a challenge even for equilibrium thermodynamics, and additional approximations are inevitable when non-equilibrium transport processes are involved. Here, we use a somewhat simplified *t*-matrix theory at low temperatures, which reports the second order superfluid phase transition and is consistent with the BCS-Leggett ground state, sometimes known as the ‘pseudogap model’^3^. We summarize the details in the Supplementary Information, and it is worth pointing out here that it captures the essential distinction between the excitation gap $\Delta$ and the superconducting order parameter $\Delta _{sc}$, with $\Delta ^2=\Delta _{sc}^2+\Delta _{pg}^2$. In other words, the contributions of the non-condensed pairs are integrated in $\Delta _{pg}$. Using the same spirit of the approximations in the correlation functions, and according to^25^, the effect of the interaction term in the heat current is relatively small at low temperatures, the final expressions of the four static coefficients are

4 $$\begin{aligned} L_{ij}=\frac{-T}{3\pi ^2m^2}\int _0^\infty dkk^4\xi _{{\mathbf{k}}}^{i+j-2} \int _{-\infty }^{\infty }\frac{d\epsilon }{4\pi }\frac{\partial f^0(\epsilon )}{\partial \epsilon }[A^2({\mathbf{k}},\epsilon ) +B_{sc}^2({\mathbf{k}},\epsilon )-B_{pg}^2({\mathbf{k}},\epsilon )], \end{aligned}$$

with the Fermi distribution $f^0(\epsilon )=(e^{\epsilon /T}+1)^{-1}$ and the spectral functions $A({\mathbf{k}},\epsilon )\!=\!-2\mathrm{Im}G({\mathbf{k}},\epsilon )$ and $B_{sc(pg)}({\mathbf{k}},\epsilon )\!=\!-2\mathrm{Im}F_{sc(pg)}({\mathbf{k}},\epsilon )$. The detailed derivation of the above expressions and the explicit forms of the generalized normal and anomalous Green’s functions $G({\mathbf{k}},\omega )$ and $F_{sc(pg)}({\mathbf{k}},\omega )$ can all be seen in the Supplementary Information. We also evaluate a temperature and interaction dependent damping term associated with the finite-lifetime effects of thermally excited carriers (relaxation time $\tau$) in the SI.

### Numerical results and comparison with experiment

**Figure 1:**
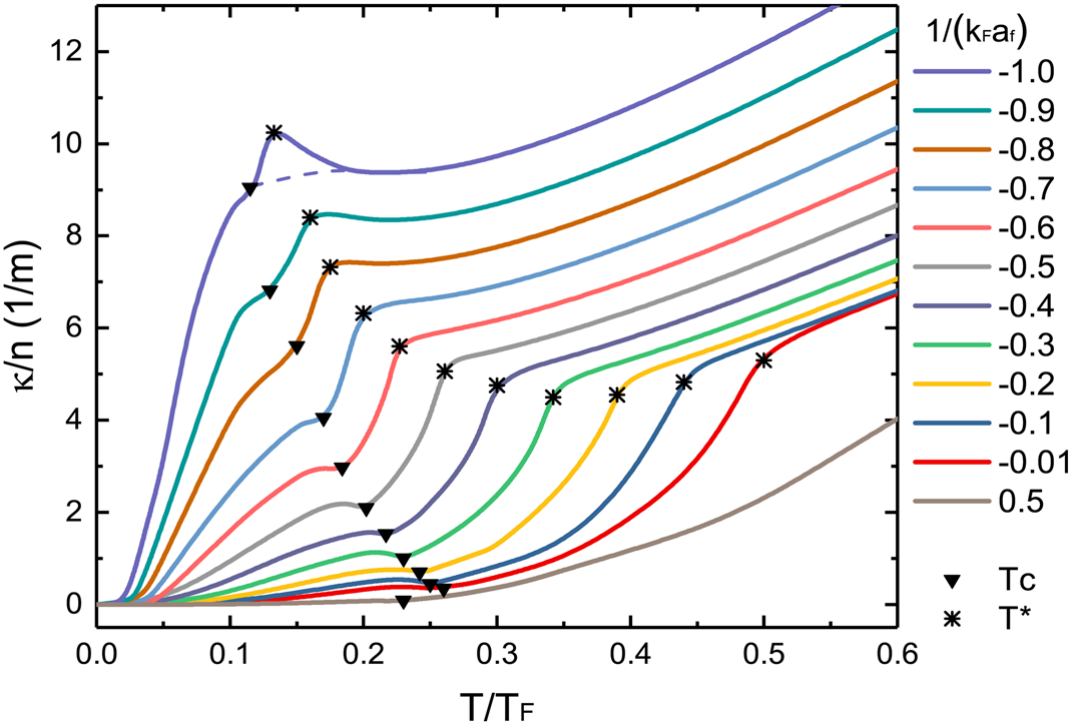
Thermal conductivity $\kappa$ of an ultracold Fermi gas, normalized by *n* and in units of 1/*m*, as a function of $T/T_F$ for different $\nu =(k_Fa_f)^{-1}$. The asterisks and triangles mark the specific thermal conductivities at $T^*$ and $T_c$, respectively. The dashed line is a possible estimate when $T^*$ is not clearly defined (see text).

Figure 1 shows the temperature dependence of calculated thermal conductivity $\kappa$ in the BCS-BEC crossover for different $\nu =(k_Fa_f)^{-1}$, the dimensionless interaction strengths with Fermi momentum $k_F$. The values of $T_c$ and $T^*$ are different for each $\nu$ and they both decrease exponentially approaching the BCS limit. In the pseudogap model, $T_c$ is obtained with the condition $\Delta _{sc}=0$ while $T^*$ from the condition $\Delta =0$. One should note that this microscopic theory yields different $T_c$ to experiments in the crossover regions. For example, at unitary limit it gives $T_c\simeq 0.26T_F$^3^ that is larger than the experimental value $T_c\simeq 0.167T_F$^30^; thus a direct quantitative comparison with experiments should be made with caution. Nonetheless, the qualitative trend here is quite intuitive. For each curve, we explicitly mark $\kappa (T^*)$ and $\kappa (T_c)$ with an asterisk and a triangle, which lie roughly at a drop point and an inflection point, respectively. These two points on each curve distinguish three distinct temperature dependencies, which characterize different regions of normal, pseudogap and superfluid states.

In the weak coupling BCS regions and above $T^*$, our Kubo calculations reduce to the kinetic theory and show great consistency with the previous knowledge via the virial expansion that $\kappa$ grows as $\sim T^{1/2}$^23^. For $\nu \lesssim -0.9$, there is a local minimum at around $(0.2-0.3)T_F$ due to the Pauli blocking. Below $T^*$, $\kappa$ drops and exhibits unexpected maximums right at $T^*$, which arise from our approaches for the pseudogap with a nonzero $\Delta _{pg}$ and suggest that the formation of pairs reduces the Pauli blocking. As $\nu$ approaches the unitary limit, these maximums disappear with the increase of $T^*$. Similar drops have been reported on shear viscosity both experimentally and theoretically^8,11,31^, and can be understood as the dramatic decrease of effective many-body carrier density^7,11,32^.

It is worth commenting that the fundamental nature of pseudogap has long been a subject of controversy and remains unclear at this time. In condensed matter physics, various kinks have been observed at $T^*$ for several transport coefficients, like the electrical and Hall resistivity, where the pseudogap may be associated with broken symmetries. However, in the *s*-wave system of interest, the preformed pairs gradually appear with decreasing temperatures in the normal phase. The questions are yet to be confirmed whether they occur simultaneously with the dip structure in the single-particle spectral weight and whether there is a precise definition of $T^*$. Therefore, if the predicted maximums of $\kappa$ at the BCS regions are absent in future’s observations, but instead look like the monotonous dashed line in Fig. 1, then there may not be a definite onset $T^*$ at which the pseudogap effects occur indeed.

At near zero temperatures, $\kappa$ decrease to zero for all $\nu$ due to the exponential increase in condensed pairs that do not transfer heat. Compared to the kinetic results that based on the thermal transport of superfluid phonons^21,23^, our results are considerably higher. In the vicinity of $T_c$, the curves we compute exhibit inflection points, which are due to the greatest contributions of pair fluctuations around the phase transition points, and also reflect different microscopic properties of the superfluid and pseudogap states. The pair fluctuations are significant near the unitary limit $\nu \simeq 0$, where the curves become non-monotonic.

**Figure 2:**
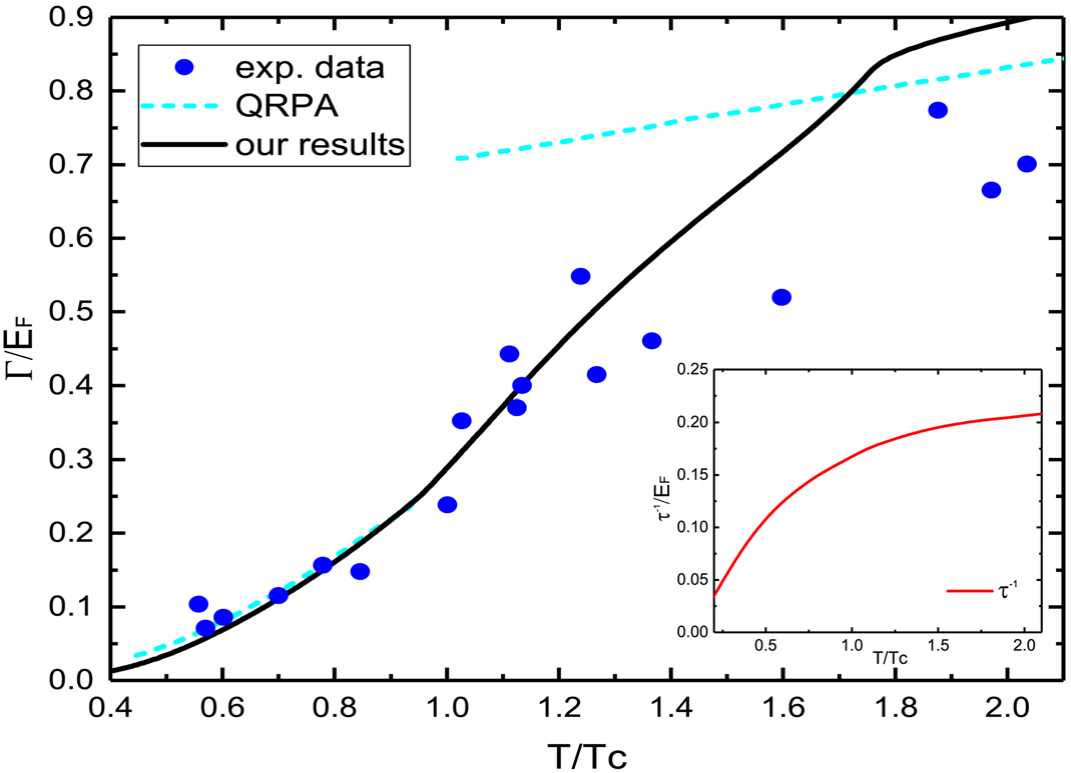
The damping rate $\Gamma$ versus $T/T_c$ at unitary limit. Black solid line is our calculations. Blue circles and the cyan dashed line are experimental data and quasiparticle random phase approximation (QRPA) theory, respectively, from^16^. Inset: the thermal relaxation rate $\tau ^{-1}$ versus $T/T_c$.

We present our estimated thermal relaxation rate $\tau ^{-1}$ (see the Supplementary Information) in units of the Fermi energy $E_F$ at the unitary limit in the inset of Fig. 2. $\tau ^{-1}$ is usually considered as an energy boundary to estimate the collisionless and hydrodynamic domains of excitation modes. Here, we find that it is smaller than the characteristic energy scales like $\mu \sim 0.4E_F$ and $\Delta \sim 0.6E_F$^30^, especially at low temperatures below $T_c$. Therefore, the system is in a weak dissipation region in the superfluid phase, which is also consistent with the studies on shear viscosity^11,21,22^. A very recent experiment on sound waves studies a similar circumstance^16^. It observes excitation modes at frequency $\omega _0\sim (0.35-0.5)E_F$, which lie in the collisionless regime in superfluid phase and the crossover between the hydrodynamic and collisionless regime above $T_c$. For these excitation modes with frequencies slightly deviating from the low-frequency limit, we consider that the hydrodynamic expressions are still approximately true. We can thus compare the damping rate $\Gamma$ with experimental data, which can be directly measured by density response and includes the contributions of shear viscosity $\eta$ and thermal conductivity $\kappa$ at unitary, as^33^

5 $$\begin{aligned} \Gamma =\left( \frac{4\eta }{3mn}+\frac{4\kappa T}{15\mathscr {P}}\right) q^2. \end{aligned}$$

Note that within the pseudogap theory, the dispersion for Goldstone bosons is quadratic in superfluid phase. Here we use the same wave vector $q=0.5k_F$ as the measurement. For $\eta$, we use our previous calculations which fit well with experiments and other theories^11^, and for the pressure $\mathscr {P}$ we use the MIT experimental data^30^. In Fig. 2, our results of $\Gamma$ as a function of $T/T_c$ are in good agreement with the experimental data near and below $T_c$. A quasiparticle random phase approximation (QRPA) calculation based on the collisions between fermions is also consistent with our results below $T_c$, which confirms that the fermionic quasiparticles are the dominant thermal excitations below $T_c$, which could also be mapped to similar cases of weak dissipation. Meanwhile, since the pseudogap theory holds that bosonic degrees of freedom contribute approximately in the near-zero range of momentum and energy^3^, the interactions between pairs can be ignored in the collisionless regime with a wave vector at $q\sim 0.5k_F$. Thus our treatment of ignoring the interaction term in the heat current operator is reasonable in this case. We can conclude that in the weak dissipation and collisionless regimes, the fermionic quasiparticles dominate in the thermal transport.

Our calculations deviate somewhat from the experimental data at higher temperatures above $T_c$, where the system can no longer be described as collisionless and has relatively strong dissipation. At this point, the large-momentum bosonic excitations play an increasingly important role, making the pseudogap theory less reliable. The scattering channel via fermionic and bosonic quasiparticles becomes more and more important^11^, which needs to be taken into account in evaluating the thermal relaxation time $\tau$. It will reduce $\tau$ and the damping rate $\Gamma$.

**Figure 3:**
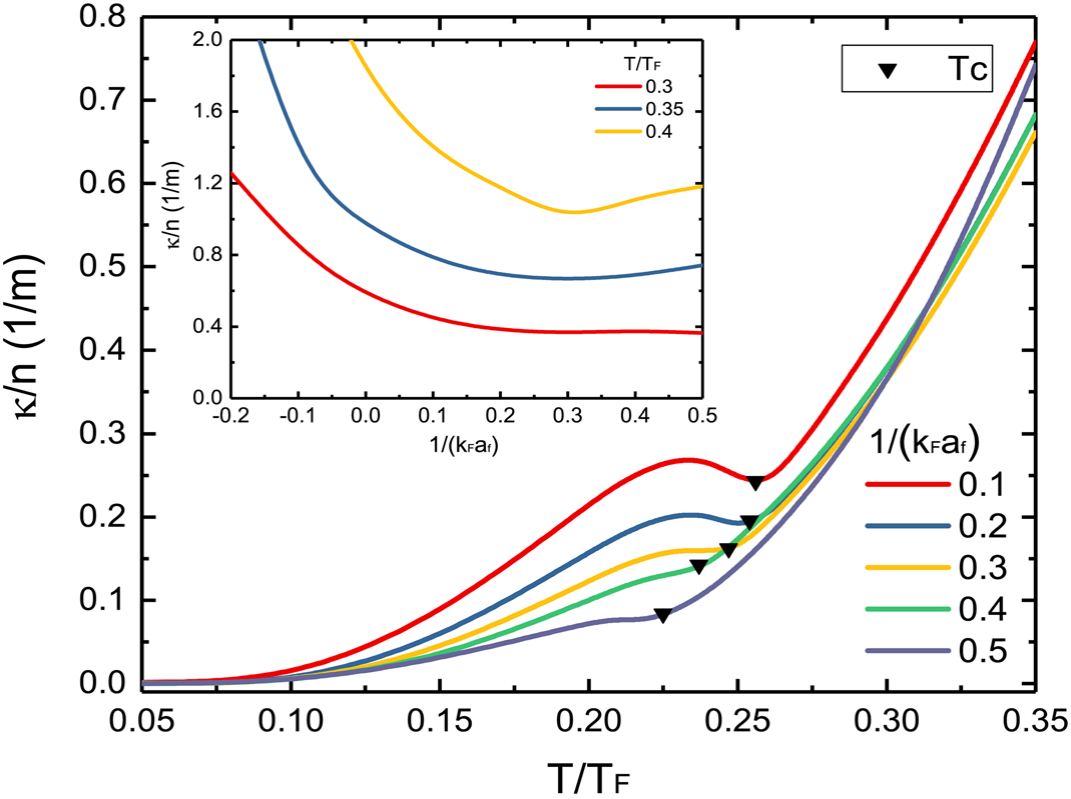
$\kappa$ versus $T/T_F$ for different $\nu$ at the BEC side. The triangles mark $\kappa (T_c)$. Here $T^*$ on each of these curves is higher than the temperature range shown. Inset: $\kappa$ versus $\nu$ for different $T/T_F$.

On the BEC side, our calculations give relatively small values of $\kappa$, the details are shown in Fig. 3. We find that the curves intersect with different interaction strength at $T\gtrsim 0.3T_F$ ($T_c\simeq 0.25T_F$, see the black triangles). This means that as a function of $\nu$, $\kappa$ exhibits a minimum at $\nu \simeq 0.3$ above the superfluid phase, as shown in the inset of Fig. 3. An anomalous minimum was found in the measurement of $\eta$ above $T_c$ at $\nu \simeq 0.25$^34^, which should have occurred at the unitary limit^12^. This minimum shift in $\eta$ can be understood by higher-order modifications in the kinetic theory^35^. Since the damping rate $\Gamma$ includes both contributions of $\eta$ and $\kappa$, we can predict that there may be a minimum on the damping rate $\Gamma$ at the wave vector $q\sim 0.5k_F$, at the interaction strength $\nu \sim (0.2-0.3)$ on the BEC side.

Since our calculations are constructed on the essential many-body fermionic natures, it is better not to span the boundary $\nu \approx 0.5$ where the zero-temperature chemical potential $\mu$ changes sign that signals the disappearing of the underlying Fermi surface and the Fermi statistics. Afterwards the system enters the two-body molecular regime where the dominant damping mechanism becomes the bosonic excitations and the thermal conductivities are expected as the almost Bose results^26,36^.

## Discussion

In summary, we have given a Kubo-based calculation for the thermal conductivity of an ultracold Fermi gas across the BCS-BEC crossover. Based on the pseudogap theory, our calculation addresses into the superfluid phase, which gives higher results of $\kappa$ than the kinetic calculations based on phonons. At high temperatures our expression of $\kappa$ reduces automatically to the Boltzmann results. We consider primarily the fermionic contributions to the thermal transport, which may be the dominant thermal carriers in the weak dissipation regions where the relaxation rate of the system is relatively smaller than the characteristic energy scales. The intrinsic physical mechanism is similar to the sound modes in the collisionless dynamic regime, and our results fit well with a recent measurement on the damping rate that contains the thermal and viscous contributions.

Our Kubo approach interpolates smoothly from the weakly dissipated low and high temperatures into the strongly dissipated pseudogap states where the relaxation rate is comparable to the characteristic energy scales of the system. The strong pairing fluctuations reduce the thermal fermionic quasiparticles and cause the thermal conductivity curve to have different temperature dependencies in different microscopic states. In these strongly dissipated regions, since the scattering between fermions is no longer the only major relaxation mechanism, more efforts can be done to incorporate other scattering channels in the future. Moreover, the interactions between pairs may need to be taken into account to obtain a complete description of thermal transport, which means to introduce an extra vertex correction term in the correlation functions^37^. A nearly simultaneous study using the Luttinger-Ward approach investigates $\kappa$ in the normal phase, which finds that the bosonic correlations are important near unitary limit above $T_c$^28^.

## Supplementary Information

Supplementary information.
