# Supplementary material for: Thermal conductivity of an ultracold Fermi gas in the BCS-BEC crossover
Source: Sci Rep. 2021 Jan 13;11:1228. doi: 10.1038/s41598-020-79010-w (PMC7806942; doi:10.1038/s41598-020-79010-w)
Supplement: Supplementary file 1 — Supplementary information. [file 41598_2020_79010_MOESM1_ESM.pdf]

Supplementary Information: Thermal conductivity of an ultracold Fermi gas in the BCS-BEC crossover

Hang Zhou, Yongli Ma
State Key Laboratory of Surface Physics and Department of Physics, Fudan University, Shanghai 200433, China

I. KUBO FORMULA AND CORRELATION FUNCTIONS

As described in the main text, we choose the specific form of concentration gradient $\mathbf{X}_1 = -\nabla(\frac{\mu}{T})$ and temperature gradient $\mathbf{X}_2 = \nabla(\frac{1}{T})$ to linearly connect the particle ($\mathbf{J}_1$) and heat ($\mathbf{J}_2$) currents in a given neutral Fermi atomic system, with $\mathbf{J}_i = \sum_{j=1}^{2} L_{ij}\mathbf{X}_j$. Considering the definition $\mathbf{J}_2 = -\kappa\nabla T$ and the measuring condition $\mathbf{J}_1 = \mathbf{0}$, the four coefficients L_{ij} $(i, j = 1, 2)$ are related to the thermal conductivity κ by [1]

$$\kappa = \frac{1}{T^2}\left(L_{22} - \frac{L_{12}L_{21}}{L_{11}}\right). \tag{1}$$

When it comes to heat transport, the entropy generation can be the linear perturbation of the system with the additional energy H', i.e., $\frac{\partial S}{\partial t} = \sum_i \mathbf{J}_i \cdot \mathbf{X}_i$ and $H'(t) = TS'(t) = \frac{\mathrm{i}T}{\Omega}\sum_i \int d\mathbf{r}\mathbf{J}_i(\mathbf{r}, t)\cdot\mathbf{X}_i(\mathbf{r})e^{-\mathrm{i}\Omega t}e^{0^+t}$. Linear response theory evaluates the measurable currents $\mathbf{J}_i$ by the thermal expectation value ($\langle\cdots\rangle$) of its operator $\mathbf{j}_i$ that

$$\mathbf{J}_i = \langle\mathbf{j}_i(\mathbf{r}, t)\rangle = \frac{T}{\Omega}\sum_j \int d\mathbf{r}' \int_{-\infty}^{+\infty} dt'\Theta(t-t')\langle[\mathbf{j}_i(\mathbf{r}, t), \mathbf{j}_j(\mathbf{r}', t')]\rangle \cdot \mathbf{X}_j(\mathbf{r}', t'), \tag{2}$$

where the step function $\Theta(t - t')$ enforces the causality. The retarded current-current correlation functions are defined by $\overleftrightarrow{L}_{ij}(\mathbf{r} - \mathbf{r}', t - t') = \mathrm{i}\Theta(t - t')\langle[\mathbf{j}_i(\mathbf{r}, t), \mathbf{j}_j(\mathbf{r}', t')]\rangle$, which are more convenient to calculate in the corresponding Matsubara formalism in momentum space

$$\overleftrightarrow{L}_{ij}(\mathbf{q}, \mathrm{i}\Omega_m) = \int_0^{1/T} d\tau e^{\mathrm{i}\Omega_m\tau}\langle T_\tau \mathbf{j}_i(\mathbf{q}, \tau)\mathbf{j}_j(-\mathbf{q}, 0)\rangle, \tag{3}$$

with $\tau = \mathrm{i}t$ and the time order operator T_τ.

Since κ is from the longitudinal response, the static coefficients L_{ij} are expressed in terms of correlation functions with the analytical continuation $\mathrm{i}\Omega_m \to \Omega + \mathrm{i}0^+$:

$$L_{ij} = -\lim_{\Omega\to 0}\frac{T}{\Omega}\lim_{\mathbf{q}\to 0}\mathrm{Im}\left(\frac{\mathbf{q}\cdot\overleftrightarrow{L}_{ij}(\mathbf{q}, \Omega)\cdot\mathbf{q}}{q^2}\right). \tag{4}$$

In the BCS-BEC crossover scenario, the Hamiltonian with zero-range interactions is

$$\hat{H} = \sum_{\mathbf{k}\sigma}\xi_{\mathbf{k}}\hat{c}^\dagger_{\mathbf{k}\sigma}\hat{c}_{\mathbf{k}\sigma} + \frac{g}{2}\sum_{\mathbf{k}\mathbf{k}'\mathbf{q}\sigma\sigma'}\hat{c}^\dagger_{\mathbf{k}+\mathbf{q}\sigma}\hat{c}^\dagger_{\mathbf{k}'-\mathbf{q}\sigma'}\hat{c}_{\mathbf{k}'\sigma'}\hat{c}_{\mathbf{k}\sigma}. \tag{5}$$

Here $\xi_{\mathbf{k}} = \mathbf{k}^2/2m - \mu$ is the dispersion measured from the chemical potential μ, m is the atomic mass and $\hat{c}^\dagger_{\mathbf{k}\sigma}(\hat{c}_{\mathbf{k}\sigma})$ is the fermion creation(annihilation) operator with the pseudospin σ. g is the bare s-wave interaction strength associated with the tunable scattering length a_f, given by $\frac{1}{g} = \frac{m}{4\pi a_f} - \sum_{\mathbf{k}}\frac{m}{\mathbf{k}^2}$. The current operators for the Hamiltonian have the form [2, 3]

$$\begin{aligned}
\mathbf{j}_1(\mathbf{q}, \tau) =& \frac{1}{2m}\sum_{\mathbf{k}\sigma}(2\mathbf{k} + \mathbf{q})\hat{c}^\dagger_{\mathbf{k}\sigma}(\tau)\hat{c}_{\mathbf{k}+\mathbf{q}\sigma}(\tau), \\
\mathbf{j}_2(\mathbf{q}, \tau) =& \frac{1}{2m}\sum_{\mathbf{k}\sigma}[\mathbf{k}\xi_{\mathbf{k}+\mathbf{q}} + (\mathbf{k}+\mathbf{q})\xi_{\mathbf{k}}]\hat{c}^\dagger_{\mathbf{k}\sigma}(\tau)\hat{c}_{\mathbf{k}+\mathbf{q}\sigma}(\tau) \\
&+ g\sum_{\mathbf{k}\mathbf{k}'\mathbf{q}'}\frac{\mathbf{q}'}{m}\hat{c}^\dagger_{\mathbf{k}+\mathbf{q}+\mathbf{q}'\uparrow}(\tau)\hat{c}^\dagger_{\mathbf{k}'-\mathbf{q}'\downarrow}(\tau)\hat{c}_{\mathbf{k}'\downarrow}(\tau)\hat{c}_{\mathbf{k}\uparrow}(\tau).
\end{aligned} \tag{6}$$

In addition, the imaginary-time single-particle Green's function is defined by $G(\mathbf{k}, \tau) = -\langle T_\tau\hat{c}_{\mathbf{k}\sigma}(\tau)\hat{c}^\dagger_{\mathbf{k}\sigma}(0)\rangle$. There are at least five kinds of $(G_\alpha G_\beta)G_\gamma$ versions of t-matrix theories that have been adopted in the normal state to evaluate the Green's function

[4]. In this work we choose the asymmetric t-matrix form called the $(GG_0)G_0$ scheme, which can be easily extended to the superfluid phase and is consistent with the BCS-Leggett ground state [5].

Each of the four correlation functions (3) can be represented as a bubble diagram in terms of the product of two Green's functions GG and a full vertex function Γ_j:

$$\overleftrightarrow{L}_{ij}(Q) = \sum_K \Gamma_i^0(K, K+Q)G(K)G(K+Q)\Gamma_j(K+Q, K), \tag{7}$$

where we have used the four-vector notation $K = (\mathbf{k}, \mathrm{i}\omega_n)$, $Q = (\mathbf{q}, \mathrm{i}\Omega_m)$ and $\sum_{K(Q)} = T\sum_{\mathrm{i}\omega_n(\Omega_m)}\sum_{\mathbf{k}(\mathbf{q})}$. Here the bare vertex factors are $\Gamma_1^0(K, K+Q) = (2\mathbf{k}+\mathbf{q})/(2m)$ and $\Gamma_2^0(K, K+Q) = [\mathbf{k}\xi_{\mathbf{k}+\mathbf{q}} + (\mathbf{k}+\mathbf{q})\xi_{\mathbf{k}}]/(2m)$, and the dressed vertex functions can be given by an integral equation

$$\begin{aligned}\Gamma_i(K+Q, K) = \Gamma_i^0(K+Q, K) + \sum_P \Lambda(K, Q, P)G(P-K) \\ \times G(P-K-Q)\Gamma_i(P-K-Q, P-K).\end{aligned} \tag{8}$$

The total irreducible contributions of interactions between the two Green's functions $\Lambda(K, Q, P)$ can be evaluated in the t-matrix level of $(GG_0)G_0$ scheme, $\Lambda(K, Q, P) \equiv t(P)$, where the t-matrix $t(Q)^{-1} = \frac{1}{g} + \sum_K G(K)G_0(Q-K)$ is on the same footing of the self energy $\Sigma(K) = \sum_Q t(Q)G_0(Q-K)$ to satisfy the Ward identities and conservation laws [6, 7]. The bare Green's function $G_0(K) = (\xi_{\mathbf{k}} - \mathrm{i}\omega_n)^{-1}$ constructs $G(K)$ through the Dyson equation $G^{-1}(K) = G_0^{-1}(K) - \Sigma(K)$.

We are interested in the low temperatures, where the effect of the interaction term in the heat current is relatively small according to [8]. In the following we consider only the contributions of four operators in the correlation functions and ignore other higher-order terms. Then to the leading order of the full vertex Γ_i, there are three types of ladder diagrams within the $(GG_0)G_0$ scheme [7]: a direct contribution called Maki-Thompson (MT) diagram and two exchange contributions called Aslamazov-Larkin (AL) diagrams, $\Gamma_i = \Gamma_i^0 + \Gamma_i^{MT} + \Gamma_i^{AL1} + \Gamma_i^{AL2}$.

II. PSEUDOGAP MODEL AND STATIC COEFFICIENTS

We adopt further simplifications of $(GG_0)G_0$ theory by the tractable 'pseudogap model' [5], which captures the essential distinction between the excitation gap Δ and the superconducting order parameter Δ_{sc} in the BCS-BEC crossover with $\Delta^2 = \Delta_{sc}^2 + \Delta_{pg}^2$, especially at the low temperatures. As a result $t(Q)$ is decomposed into a standard BCS term $t_{sc}(Q) = -(\Delta_{sc}^2/T)\delta(Q)$ and the remaining nonzero momentum pairs, which lead to the pseudogap effects and have the definition $\Delta_{pg}^2 \equiv -\sum_{Q\neq 0} t_{pg}(Q)$.

In the same spirit of these approximations, the vertex function Γ_i can also be decomposed into the superconducting $\Gamma_{i(sc)}$ and pseudogap $\Gamma_{i(pg)}$ contributions, where the former contains only the MT diagram and the latter with all the MT and AL diagrams can be further simplified to $\Gamma_i = \Gamma_i^0 - \Gamma_i^{MT}$ [7]. We then insert Eq. (8) into Eq. (7), it turns to a compact form

$$\begin{aligned}\overleftrightarrow{L}_{ij}(Q) = \sum_K \Gamma_i^0(K, K+Q)\Gamma_j^0(K+Q, K)[G(K)G(K+Q) \\ + F_{sc}(K)F_{sc}(K+Q) - F_{pg}(K)F_{pg}(K+Q)].\end{aligned} \tag{9}$$

After doing the frequency summations and taking the two limits in (4), we arrive the four static coefficients at

$$L_{ij} = \frac{-T}{3\pi^2 m^2}\int_0^\infty dk k^4 \xi_{\mathbf{k}}^{i+j-2}\int_{-\infty}^\infty \frac{d\epsilon}{4\pi}\frac{\partial f^0(\epsilon)}{\partial\epsilon}[A^2(\mathbf{k},\epsilon) + B_{sc}^2(\mathbf{k},\epsilon) - B_{pg}^2(\mathbf{k},\epsilon)], \tag{10}$$

with the Fermi distribution $f^0(\epsilon) = (e^{\epsilon/T}+1)^{-1}$ and the spectral functions $A(\mathbf{k},\epsilon) = -2\mathrm{Im}G(\mathbf{k},\epsilon)$ and $B_{sc(pg)}(\mathbf{k},\epsilon) = -2\mathrm{Im}F_{sc(pg)}(\mathbf{k},\epsilon)$. The generalized normal and anomalous Green's functions are the same as those pseudogap model and some high-T_c literatures [9, 10]

$$\begin{aligned}G(\mathbf{k},\omega) &= \left(\omega - \xi_{\mathbf{k}} + \mathrm{i}\gamma - \frac{\Delta_{pg}^2}{\omega+\xi_{\mathbf{k}}+\mathrm{i}\gamma} - \frac{\Delta_{sc}^2}{\omega+\xi_{\mathbf{k}}}\right)^{-1}, \\ F_{sc}(\mathbf{k},\omega) &= \frac{-\Delta_{sc}}{\omega+\xi_{\mathbf{k}}+\mathrm{i}0^+}\frac{1}{\omega-\xi_{\mathbf{k}}-\frac{\Delta^2}{\omega+\xi_{\mathbf{k}}}+\mathrm{i}0^+}, \\ F_{pg}(\mathbf{k},\omega) &= \frac{-\Delta_{pg}}{\omega+\xi_{\mathbf{k}}+\mathrm{i}\gamma}G(\mathbf{k},\omega).\end{aligned} \tag{11}$$

Here the additional term $\gamma = (2\tau)^{-1}$ distinguishes the non-condensed pairs by their incoherent signatures and contributes to the normal state broadenings of excitation spectra. This damping term is temperature and interaction dependent, associated with the finite-lifetime effects of thermally excited carriers.

III. THE THERMAL RELAXATION TIME AND HIGH TEMPERATURE GENERALIZATION

The transport properties of a system are controlled by the longest-lived excitations. In a weak dissipated system, equilibration is slow and the lifetime τ of quasiparticles is long. This circumstance is fulfilled at high and low temperature limits for strongly interacting Fermi gases and one may obtain the lifetime or thermal relaxation time τ through Boltzmann equation with high precision, which consists with the large-N and strong-coupling Luttinger-Ward results [3, 11]. At moderate temperatures near T_c, however, the intrinsic timescale τ is reduced by pairing fluctuations and the system enters a strong dissipation regime. According to a careful comparison of various elastic and inelastic scattering channels for fermionic and bosonic degrees of freedom [12], the scattering between unpaired fermions in this temperature region is the dominant damping mechanism, and the lifetime of fermions is considerably longer than the non-condensed pairs. It suggests that τ can be estimated approximately via scatterings with fermionic excitations.

Below T^*, the particle number conservation relation holds as $n = n_f + 2n_b$, where n is the total density and n_f and n_b denote the density of unpaired fermions and Fermi pairs, respectively. From the microscopic pseudogap theory, one can distinguish these components (see, e.g. [5, 12]), and here we only focus on the unpaired fermions $n_f = 2\sum_{\mathbf{k}} f^0(\xi_{\mathbf{k}})$ which is different from the total atomic density $n = 2\sum_K G(K)$. The distribution of the fermions is assumed to change linearly in thermal transport processes as $\delta f = f^0(1-f^0)\Phi$. The trial function Φ is chosen as $\Phi(\mathbf{k}) = \frac{\mathbf{k}}{m}[\frac{k^2}{2mT} - \frac{5f_{5/2}(z)}{2f_{3/2}(z)}]$ [13], where $z = e^{\mu/T}$ is the fugacity and $f_\alpha(z) = \frac{1}{\Gamma(\alpha)}\int_0^\infty \frac{x^{\alpha-1}dx}{z^{-1}e^x+1}$ are the Fermi functions. The thermal relaxation rate can be evaluated by Boltzmann equation under relaxation time approximation

$$\frac{1}{\tau} = \frac{-\sum_{\mathbf{k}} \Phi^* C[\Phi]}{\sum_{\mathbf{k}} |\Phi|^2 f^0(1-f^0)} = \frac{16mT^2 I_\tau(z)}{15\pi^{9/2} D_\tau(z)}. \tag{12}$$

Here the collision integral $C[\Phi]$ has the same form as [13], $D_\tau(z) = \frac{7}{2}f_{7/2}(z) - \frac{5f_{5/2}^2(z)}{2f_{3/2}(z)}$ and

$$\begin{aligned} I_\tau(z) =& \sqrt{2}\pi^3 \int_0^\infty dx_0 \int_0^\infty dx_r \int_{-1}^1 dy \int_{-1}^1 dy' x_0^4 x_r^7 \\ & \times (y^2 + y'^2 - 2y^2 y'^2) \frac{d\sigma_f}{d\Omega} F(x_0, x_r, y, y'), \end{aligned} \tag{13}$$

with $F = f_1^0 f_2^0 (1-f_3^0)(1-f_4^0)$ denoting the probability production of ingoing and outgoing particles. The differential cross section is $\frac{d\sigma_f}{d\Omega} = \frac{a_f^2}{1+k_r^2 a_f^2}$ with the relative momentum $\mathbf{k}_r = (\mathbf{k}_1 - \mathbf{k}_2)/2$ and the variable substitution $k_r = \sqrt{2mTx_r}$.

It might seem strange to apply a kinetic evaluation below T^*. We point out again that the microscopically dominant strong correlated nature has been carried by this scattering channel after a comprehensive analysis of the various channels among various excitations. Meanwhile, in our interested low temperatures, there is an exponential decay factor $e^{-a/T}$ that predominates in the expression of κ in Eq. (10) and makes the quantitative values of τ less important. And a literature on viscosity found the final results quite independent of τ by trying different values of it [14].

It is instructive to derive the kinetic expression from our Kubo formula (7) in high-T limit. To see this, the Green's function has the form $G(K) = (\mathrm{i}\omega_n - \xi_{\mathbf{k}} + \mathrm{i}\gamma)^{-1}$, and with a vanishing peak width $\gamma \approx 0$ in the weak dissipation regime, most of the spectral weight concentrates in the narrow peak $A^2(\mathbf{k},\epsilon) \to 2\pi\delta(\epsilon - \xi_{\mathbf{k}})/\gamma$. These on-shell fermions imply the ignorable medium effects induced by pairs, as a result the $t-$matrix reduces to the vacuum scattering amplitude $t(Q) \to 4\pi a_f/[m(1+\mathrm{i}qa_f)]$ and the vertex corrections approach zero. Thus Eq. (10) reduces to $L_{ij} = \frac{T\tau I_{ij}}{3\pi^2 m^2}$ with $I_{ij} = -\int_0^\infty dk k^4 \xi_{\mathbf{k}}^{i+j-2} \frac{\partial f^0(\xi_{\mathbf{k}})}{\partial \xi_{\mathbf{k}}}$, and consequently $\kappa = \frac{\tau}{3\pi^2 m^2 T}(I_{22} - \frac{I_{12}I_{21}}{I_{11}})$. Using the formula $\int dx x^{\alpha-1} f^0(\xi_{\mathbf{k}})[1-f^0(\xi_{\mathbf{k}})] = z\frac{\partial}{\partial z}[\Gamma(\alpha)f_\alpha(z)] = \Gamma(\alpha)f_{\alpha-1}(z)$, we can express the integrals I_{ij} by the Fermi functions and obtain

$$\kappa = \frac{\tau(2mT)^{5/2}\Gamma(7/2)}{6\pi^2 m^2}\left[\frac{7}{2}f_{7/2}(z) - \frac{5f_{5/2}^2(z)}{2f_{3/2}(z)}\right]. \tag{14}$$

By inserting Eq. (12) into this equation, we get

$$\kappa = \frac{75\pi^3}{64}\sqrt{\frac{2T}{m}}\frac{D_\tau^2}{I_\tau}, \tag{15}$$

consistent with the Boltzmann results derived for a Bose system in [13].

---

[1] Mahan, G. D. Many-particle physics (third edition, Plenum Publishers, New York, 2000).
[2] Fujii, K. & Nishida, Y. Hydrodynamics with spacetimedependent scattering length. *Phys. Rev. A* **98,** 063634 (2018).
[3] Frank, B., Zwerger, W. & Enss, T. Quantum critical thermal transport in the unitary Fermi gas. *Phys. Rev. Research* **2,** 023301 (2020).
[4] Pini, M., Pieri, P. & Strinati, G. C. Fermi gas throughout the BCS-BEC crossover: Comparative study of t-matrix approaches with various degrees of self-consistency. *Phys. Rev. B* **99,** 094502 (2019).
[5] Chen, Q. J., Stajic, J., Tanb, S. & Levin, K. BCS-BEC crossover: From high temperature superconductors to ultracold superfluid. *Phys. Rep.* **412,** 1 (2005).
[6] He, Y. & Levin, K. Establishing conservation laws in pair-correlated many-body theories: T-matrix approaches. *Phys. Rev. B* **89,** 035106 (2014).
[7] Kosztin, I., Chen, Q. J., Kao, Y-J. & Levin, K. Pair excitations, collective modes, and gauge invariance in the BCS-Bose-Einstein crossover scenario. *Phys. Rev. B* **61,** 11662 (2000).
[8] Kadanoff, L. P. & Martin, P. C. Theory of many-particle systems. II. Superconductivity. *Phys. Rev.* **124,** 670 (1961).
[9] Chen, Q. J., He, Y., Chien, C-C. & Levin, K. Theory of radio frequency spectroscopy experiments in ultracold Fermi gases and their relation to photoemission in the cuprates. *Rep. Prog. Phys.* **72,** 122501 (2009).
[10] Guo, H., Wulin, D., Chien, C-C. & Levin, K. Perfect fluids and bad metals: insights from ultracold Fermi gases. *New J. Phys.* **13,** 075011 (2011).
[11] Enss, T., Haussmann, R. & Zwerger, W. Viscosity and scale invariance in the unitary Fermi gas. *Ann. Phys.* **326,** 770 (2011).
[12] Zhou, H., Dong, H. & Ma, Y. L. Scattering mechanisms, relaxation times times, and shear viscosity in universal anomalous transport of unitary Fermi gases. *Phys. Rev. Research* **2,** 023166 (2020).
[13] Nikuni, T. & Griffin, A. Hydrodynamic damping in trapped Bose gases. *J. Low Temp. Phys.* **111,** 793 (1998).
[14] Guo, H., Wulin, D., Chien, C-C. & Levin, K. Microscopic approach to shear viscosities of unitary Fermi gases above and below the superfluid transition. *Phys. Rev. Lett.* **107,** 020403 (2011).
